# Applications of click and click-to-release chemistry in biomaterials to advance skin regeneration

**DOI:** 10.1039/d5cb00065c

**Published:** 2025-07-28

**Authors:** Merel Gansevoort, Matthijs van de Waarsenburg, Thomas J. Boltje, Floris P. J. T. Rutjes, Toin H. van Kuppevelt, Willeke F. Daamen

**Affiliations:** a Department of Medical BioSciences, Research Institute for Medical Innovation, Radboud University Medical Center Geert Grooteplein Zuid 10 6525 GA Nijmegen The Netherlands willeke.daamen@radboudumc.nl; b Department of Synthetic Organic Chemistry, Institute for Molecules and Materials, Radboud University Heyendaalseweg 135 6525 AJ Nijmegen The Netherlands floris.rutjes@ru.nl

## Abstract

Achieving skin regeneration following destruction of the epidermis and dermis (*e.g.* full-thickness wounds) has remained an unachieved goal. The wound healing response is complex and consists of multiple overlapping phases which are tightly choreographed by the ebb and flow of effector molecules. Mimicking this spatiotemporal aspect in pro-regenerative biomaterials may enhance their efficacy and eventually lead to skin regeneration. However, robust spatiotemporal signalling has remained difficult to achieve. The field of bioorthogonal click and click-to-release chemistry may be key to creating spatiotemporal signalling biomaterials. The ability to safely and effectively conjugate or release molecules in complex biological environments has transformed many areas of research. In this review, we aim to highlight the complex nature of wound healing and address how click and click-to-release chemistry approaches could contribute to the development of biomaterials with spatiotemporal control over effector molecules.

## Abstract 2

Challenges in wound healing may be solved using click chemistry strategies!.

## Graphical abstract

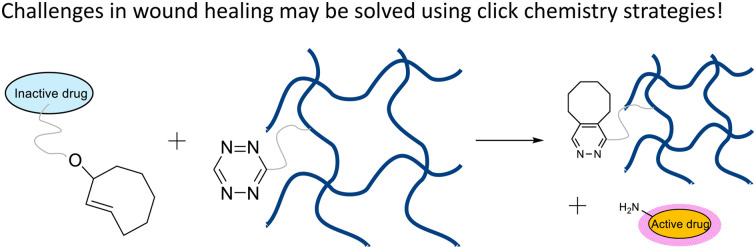

## 1. Introduction

Skin wound healing is an essential process for all mammals. As skin offers protection against all manner of harmful agents and external stimuli such as viruses, bacteria, UV light, *etc.* damage to the skin needs to be repaired quickly to maintain the barrier function. The problem addressed by this review is the limited restorative capacity of the skin. Small or surface level wounds, where only the epidermis and small parts of the underlying dermis are damaged, are able to heal without interference.^1^ However, wound healing can evolve into a fibrotic response that culminates in scar formation when the epidermis and entire dermis are damaged (full-thickness wounds). Large scars originating from this fibrotic response have a significant impact on a patient's life: they can be painful and tight, limiting the range of movement when located near joints and the disfigurements can be detrimental to mental health.^2^ The biological processes underlying wound healing and scarring are described in Section 2.

Achieving complete restoration of the skin (*i.e.* skin regeneration) after a full thickness wound is an important goal of researchers worldwide. The development of pro-regenerative biomaterials has become increasingly important in accomplishing this goal and requires the collaboration of a wide range of scientific disciplines from cell biology to chemistry. Biomaterials, which can be natural, synthetic or a combination of both, are designed to be biocompatible, *i.e.*, to safely interact with biological systems.^3^ The biomaterial itself can have a biological effect, and additional components – such as small biologically active molecules – are added to influence specific processes. In the context of wound healing, biomaterials are designed in such a way that they support the healing process and prevent/minimise scar formation.^4^ Section 3 describes the interplay between biomaterials, active molecules and wound healing, including the current limitations, in more detail. There are many options available for creating biomaterials and adding active molecules to these materials. This review focuses specifically on the biological aspects of wound healing and addresses how the chemical toolbox of bioorthogonal click and click-to-release chemistry can be harnessed to promote the development of pro-regenerative biomaterials (Sections 4 and 5).

## 2. Skin biology and the wound healing response

**Fig. 1:**
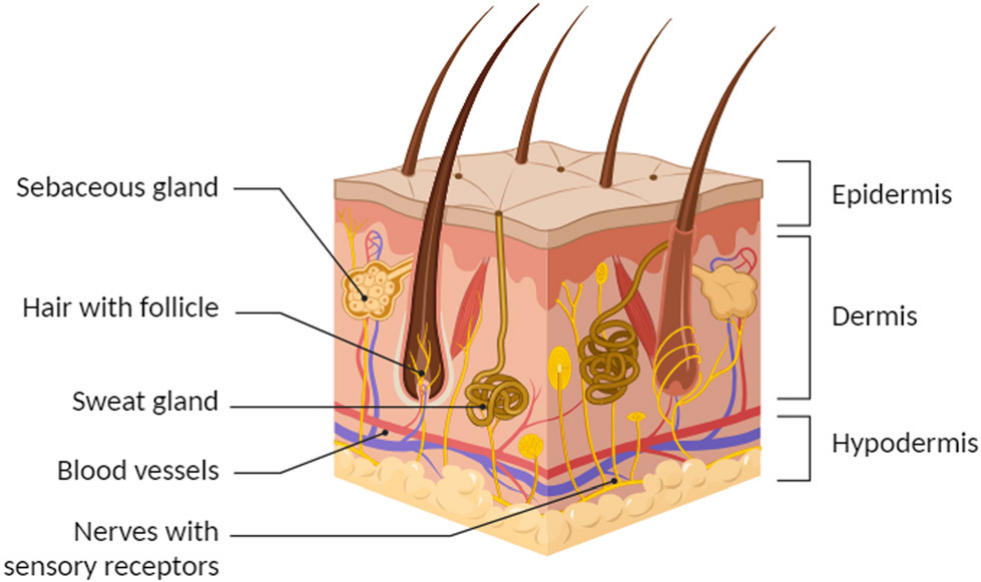
Schematic cross section of healthy skin showing the epidermis, dermis with skin appendages and including the underlying hypodermis. Created in https://BioRender.com.

The goal of skin regeneration is to restore normal skin physiology. Normal skin is divided into two layers: the epidermis and the dermis (Fig. 1). Skin appendages, such as sweat glands and hair follicles, are embedded in these layers. The epidermis is the outermost layer of the skin and consists primarily of keratinocytes interspersed with pigment-producing melanocytes, immunological cells (Langerhans cells) and Merkel cells. In the dermis, which is located below the epidermis, two distinct layers are found.^5^ The uppermost papillary layer, which forms the dermal papillae, has a loosely organised collagen-rich extracellular matrix (ECM) with a fine elastin network. The lower reticular layer is characterised by a dense network of collagen and elastin, which provides strength and elasticity. Dispersed throughout the dermis are fibroblasts, mast cells and macrophages. Blood vessels and nerves reach into the dermis from beneath the fat-rich hypodermis. In mammals the wound healing response is generally not able to restore this normal skin architecture following a full thickness wound. Instead, fibrotic skin is characterized by reduced elasticity, with the ECM containing thick bundles of type I collagen and a lack of skin appendages.^6^ This scar tissue is clearly distinguishable from the surrounding healthy skin.

**Fig. 2:**
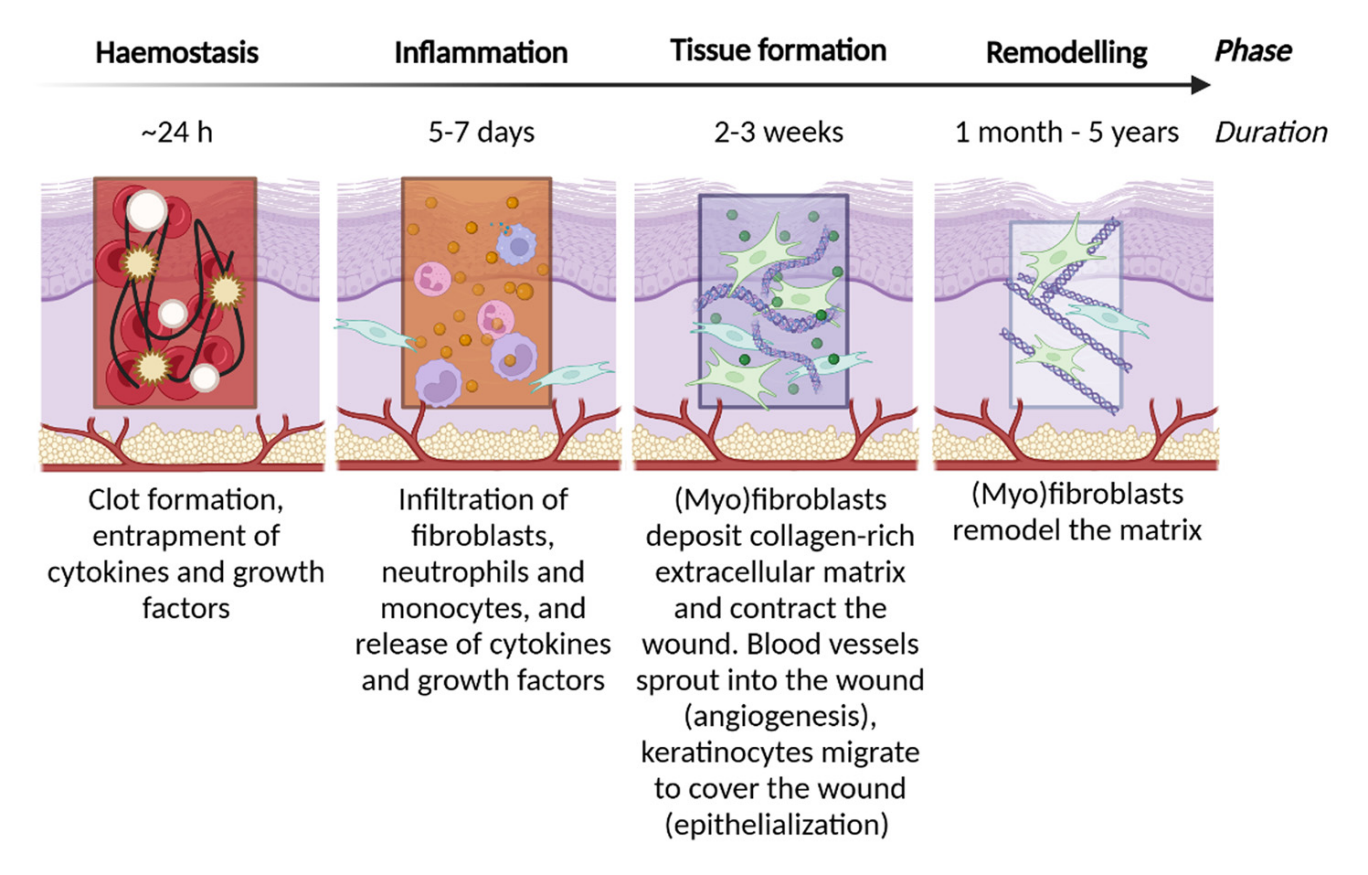
Timeline of the wound healing phases and schematic overview of activities during the wound healing phases. Created in https://BioRender.com.

Wound healing is an intricate process with four tightly choreographed phases that are constrained spatiotemporally (*i.e.*, in time and space, Fig. 2).^7^ First, a clot is formed during the haemostatic phase, this network of platelets (small cell fragments) and fibrin (a protein that forms mesh-like structures) provides a temporary matrix and prevents blood loss.^8^ This clot also captures biologically active molecules that drive the wound healing response. These include cytokines, which are small proteins of <30 kDa that act as chemical messengers between (mainly) immune cells, and growth factors: biologically active molecules secreted by cells and which influence cell growth, differentiation, and mitosis. For example, the clot captures the cytokine interleukin-1 (IL-1) and platelet derived growth factor (PDGF), which together attract neutrophils to the wound site.^9^

Monocytes and neutrophils are attracted to the wound site which signals the start of the inflammatory phase. During this approximately six-day period immune cells, macrophages (which are differentiated monocytes) and neutrophils remove pathogens and other debris, and increasing levels of cytokines and growth factors are secreted.^10^ Third is the tissue formation or proliferation phase, which starts with the activation of fibroblasts: these migrate to the wound site and differentiate into myofibroblasts under the influence of transforming growth factor β1 (TGF-β1, secreted by macrophages and platelets).^11^ These (myo)fibroblasts deposit large amounts of ECM, mainly type I collagen, and contract the wound while at the same time their continued activation (*i.e.* overproduction of ECM and wound deformation) is a major contributor to skin fibrosis.^12^ The newly deposited ECM, known as granulation tissue, will provide a basis for the remaining wound healing process. Meanwhile keratinocytes migrate to cover the wound (*i.e.* reepithelialisation) and blood vessels invade the granulation tissue (angiogenesis). Lastly, after approximately three weeks, the remodelling phase starts.^13^ This final phase can take months or years to be completed and entails the remodelling of the temporary granulation matrix by macrophages and myofibroblasts and matrix remodelling enzymes such as matrix metalloproteinases (MMP's) into the characteristic smooth scar tissue. Fibrosis can be aggravated by the dysregulation of any of the factors that are a part of the normal wound healing process, of which excessive inflammation and/or overactive myofibroblasts are the most prominent.^14^ In this regard, guiding the various wound healing stages to limit fibrosis and promote skin regeneration is a complicated endeavour.

## 3. Stimulating wound healing with biomaterials and effector molecules

The spatiotemporal nature of the wound healing response makes it a complex process to guide or to emulate using a biomaterial. Approaches that aim to promote skin regeneration range from simple wound dressings that keep the wound site moist or prevent infection, to complex skin substitutes that mimic the architecture of the epidermis and dermis to support cell ingrowth.^15,16^ Novel approaches often use a biomaterial in combination with effector molecules. These are bioactive compounds – synthetic or natural in origin – that elicit a biological response, aiming to stimulate or inhibit specific events in the wound healing cascade.^17^ The individual components of a pro-regenerative material are carefully selected to fulfil specific purposes. The biomaterial may provide mechanical stability to the damaged tissue, influence cell phenotypes or facilitate cell migration.^18^ A wide variety of materials, ranging from naturally occurring substances such as collagens, chitosan, alginate and hyaluronic acid, to synthetic polymers (*e.g.*, polyethylene glycol, polycaprolactone) and combinations thereof can be applied.^19–23^ Effector molecules, such as growth factors, cytokines or small molecules (in this review synthetic growth factor mimics in particular), have been valuable additions to biomaterials as they can control cell behaviour and help guide biological processes.^24,25^ Topical application of fibroblast growth factor 2, a potent inhibitor of the fibroblast-to-myofibroblast transition, reduced scar formation in deep paediatric hand burns.^26^ Specialised drug delivery systems have further improved the use of effector molecules: these are designed to deliver one or more effector molecules to the wound site and offer a certain amount of control over the release of the effector molecules, such as maintaining a steady release gradient or release in response to physical stimuli (such as pH or infrared light).^27^ So far, combinations of biomaterials and effector molecules have met limited clinical success. The benefits and shortcomings of these materials – as well as reasons for clinical failure – have been extensively described.^28–30^ An overarching limitation of biomaterial-effector molecule combinations remains the lack of robust spatial and temporal control over the release of effector molecules. As stated, the wound healing response is intricately orchestrated in a spatiotemporal manner. Mimicking this spatiotemporal aspect in biomaterials may improve their regenerative potential and increase the chances for successful clinical application. The delivery of effector molecules *via* biomaterials is a form of regional release, as the location of the material in the body can be controlled. Achieving spatial control within the material – for example different zones carrying different effectors – is more complex. Especially temporal control over effector molecule delivery remains challenging to accomplish. The field of bioorthogonal click and click-to-release chemistry could be essential to achieve robust spatiotemporal signalling from biomaterials and may open a new area of research with the potential to emulate complex and dynamic biological processes.

## 4. Click and click-to-release chemistry

**Fig. 3:**
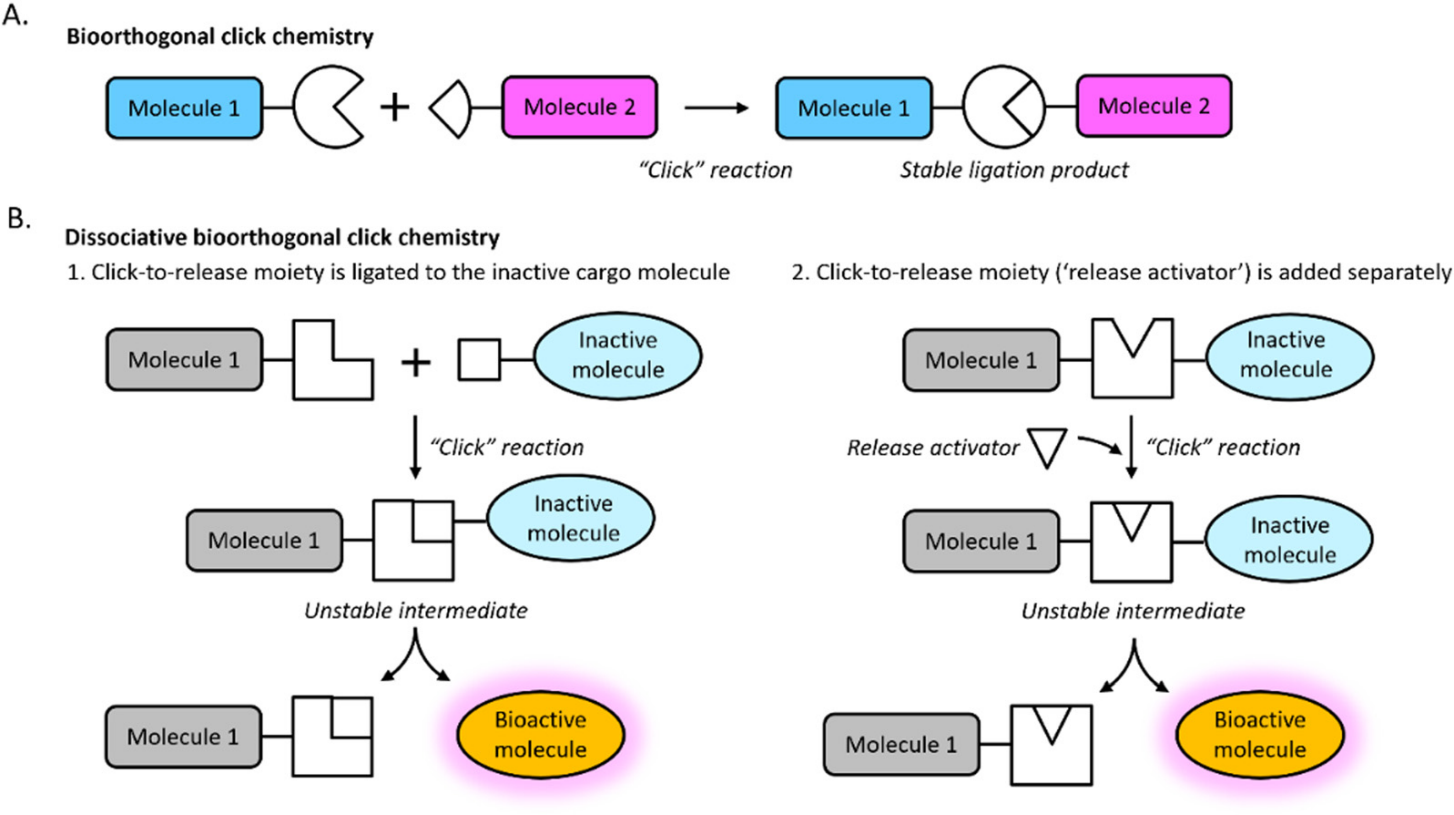
Schematic representation of bioorthogonal click chemistry approaches. (A) Bioorthogonal click reactions enable efficient and highly specific ligation of two molecules *via* click chemistry moieties that “click” together to form a stable ligation product. (B) In dissociative bioorthogonal or “click-to-release” reactions, the ligation product that forms after the click chemistry moieties have “clicked” is unstable, leading to dissociation of the bond between the click moiety and the inactive molecule. The released molecule becomes a bioactive molecule. Two options are available for dissociative reactions: the click-to-release moiety is ligated to the inactive molecule (option 1), or the click-to-release moiety (“release activator”) is added separately.

First reported in 2001 as a straightforward method for molecular synthesis,^31^ click chemistry reactions involve two – unique mutually reactive – molecular entities that ‘click’ together to form a stable covalent bond (Fig. 3A). In general, click reactions are fast, modular, simple to perform and highly specific. Some click reactions are bioorthogonal: reactions that can occur in living systems without interfering with native biochemical processes. Bioorthogonal click chemistry is gaining increasing traction in a variety of research areas, including tissue engineering and regenerative medicine.

Dissociative bioorthogonal click reactions, also known as ‘click-to-release’ reactions, were developed for the opposite purpose: to break covalent bonds to specifically release a cargo molecule in a controlled and efficient manner (Fig. 3B). In general, these reactions are fast, non-toxic, and highly specific. They are suited to *in vivo* use due to their bioorthogonal nature: the reaction components do not interfere with other biochemical processes. This makes click-to-release chemistry uniquely suitable for the development of pro-regenerative biomaterials with spatiotemporal control over effector molecules. A wide variety of click and click-to-release reactions exist, making for a versatile toolbox. The various reaction types have been described extensively by other authors.^32–36^ Therefore, this section will only provide a concise overview of the most well-known associative and dissociative bioorthogonal click chemistry reactions.

### 4.1. Staudinger reactions

**Scheme 1:**
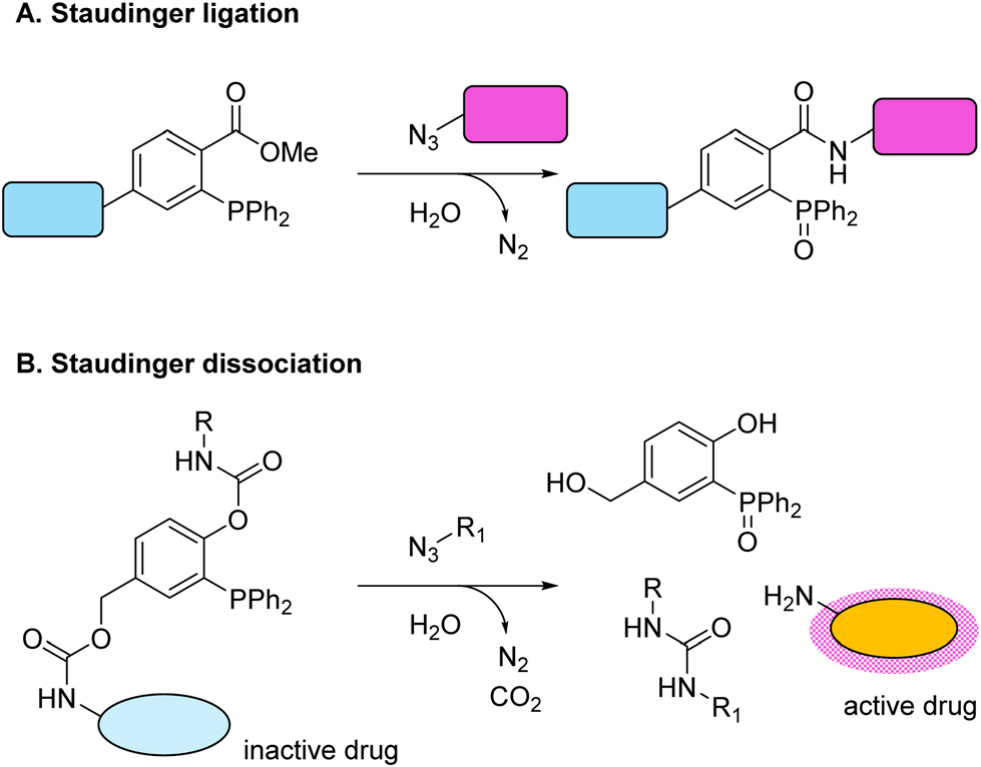
(A) The Staudinger ligation was the first-developed click reaction. This ligation reaction is often used to conjugate two molecules and occurs between a phosphine and azide group.^32,37^ (B) The Staudinger dissociation reaction was developed afterwards. Here, the phosphine moiety was modified with a carbamate group to facilitate self-immolation and allow for the release of a drug.^39^

One of the first developed bioorthogonal click reactions was the Staudinger ligation: a reaction between a phosphine and azide group resulting in an amide link (Scheme 1A).^32,37^ The original Staudinger ligation suffered from several drawbacks, including relatively slow reaction kinetics and potential oxidation of the phosphine moiety in physiological environments.^38^ The Staudinger ligation has been optimised to address these limitations, and this click reaction type has remained a valuable platform for bioconjugation. Furthermore, the Staudinger reaction has been modified to achieve dissociation instead of ligation. To achieve self-immolation, the ester group on the phosphine was replaced with a carbamate group, resulting in the formation of a relatively stable phenolate anion and eventual concomitant liberation of the bioactive molecule (Scheme 1B).^39^ The use of Staudinger dissociation reactions has been limited, but the approach has shown potential to selectively release chemotherapeutics like doxorubicin in cell culture systems.^40,41^

### 4.2. Azide–alkyne cycloadditions

**Scheme 2:**
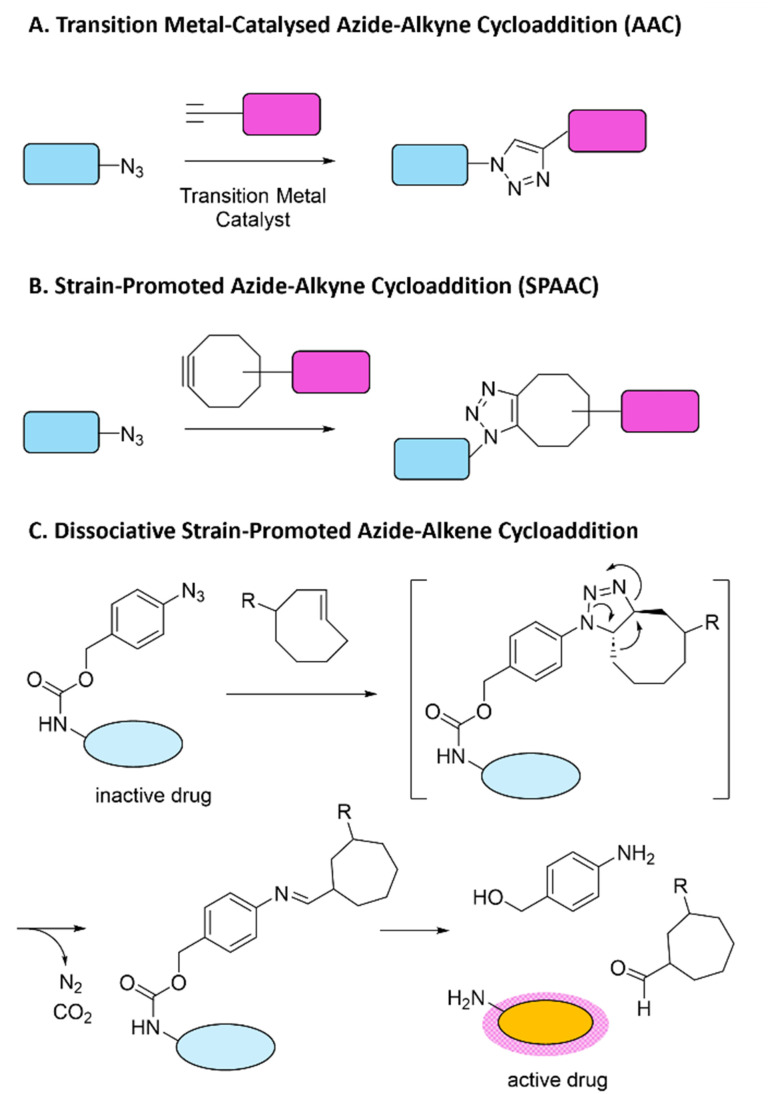
(A) The azide–alkyne (3+2) cycloaddition (AAC) was developed to achieve faster reaction kinetics. The AAC reaction results in the formation of a stable triazole bond that facilitates ligation of two molecules. The reaction kinetics can be sped up by introducing transition metals as catalysts.^42,43^ (B) In the strain-promoted azide–alkyne cycloaddition (SPAAC): functionalising the azide with a strained cyclooctyne improved the reaction kinetics without the need for transition metal catalysts.^48^ (C) Payload (drug) release *via* azide moieties can be achieved by adding a self-immolating group, subsequent reaction with an alkene then leads to release of the active drug.^33,49^

The quest for ever faster reaction kinetics led to the development of azide–alkyne cycloadditions (AAC), in which a 1,3-dipolar cycloaddition between the azide and a terminal alkyne results in the formation of a triazole bond (Scheme 2A).^42^ Initially a slow reaction, the reaction kinetics were greatly enhanced by the inclusion of a copper catalyst (CuAAC), also resulting in the sole formation of the 1,4-substituted regioisomer.^43^ Aside from copper, other transition metals can be used to catalyse AAC reactions (palladium,^44^ gold,^45^ platinum,^46^ and ruthenium^47^), in the latter case leading selectively to the 1,5-regioisomer. Overall, metal-catalysed AAC reactions have limited potential *in vivo*: the transition metals generally lead to cytotoxicity and complex molecular environments – like the ones encountered in living systems – can hinder the reactions by capturing of the metal ions.

AAC reactions were evolved further with the development of the strain-promoted azide–alkyne cycloaddition (SPAAC), where a strained cyclooctyne was introduced as an alternative to the terminal alkyne leading to significantly enhanced reaction kinetics without requiring exogenous catalysts (Scheme 2B).^48^ Thus, the SPAAC reaction mitigated the toxicity-related limitations of metal-catalysed AAC reactions and their relatively slow reaction kinetics have since the first examples been improved. Dissociation reactions involving azide groups can be achieved with strained alkenes, by adding a self-immolating linker to the azide group: this leads to release of a caged moiety as the ligation product of azides with strained cyclooctenes is unstable (Scheme 2C).^33,49^ This reaction is on the slower side and risk of hydrolysis further impacts its widespread application.

### 4.3. Inverse-electron demand Diels–Alder reactions

**Scheme 3:**
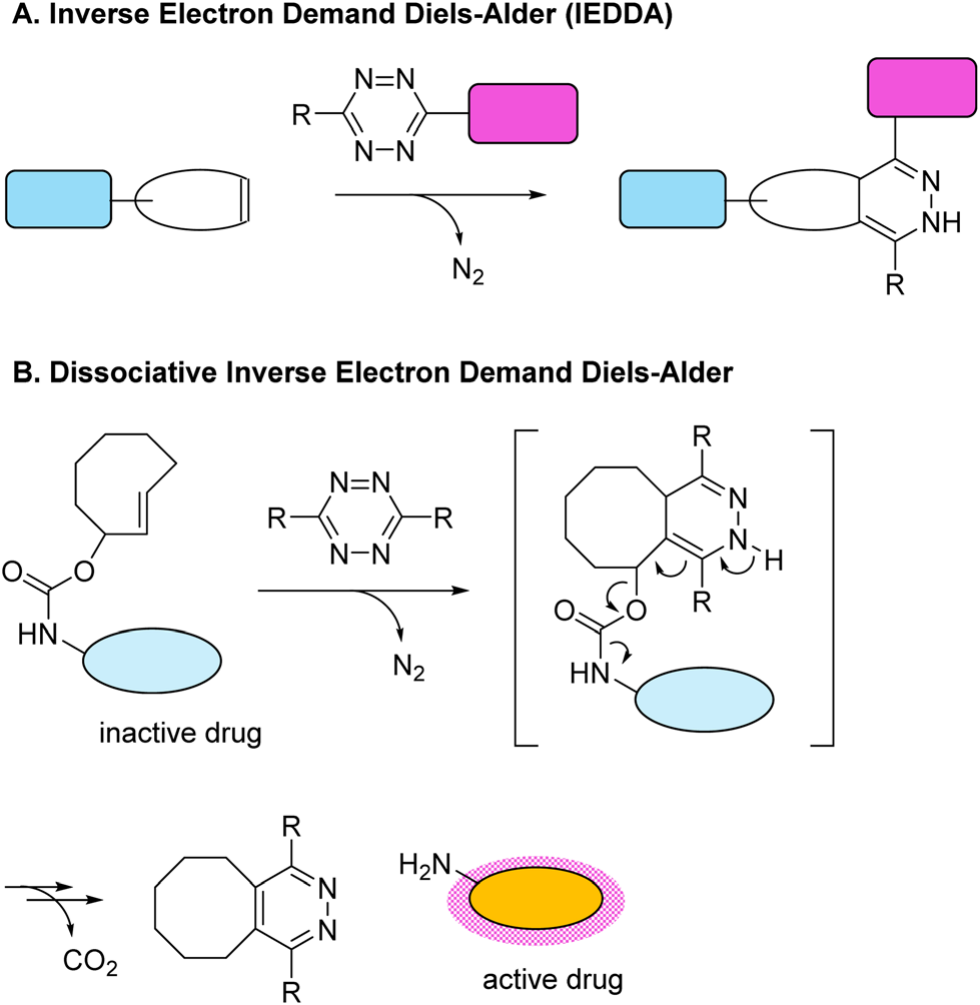
(A) The inverse electron demand Diels Alder (IEDDA) reaction was developed most recently. This reaction between dienes and dienophiles is fast, safe and efficient at ligating two molecules.^50,51^ (B) Dissociative IEDDA reactions between a *trans*-cyclooctene (TCO) and 1,2,4,5-tetrazine result in the fast release of an active drug.^31,52^

The most recently developed bioorthogonal click reaction, the inverse-electron-demand Diels–Alder (IEDDA) reaction, overcame the limitations of its predecessors.^50,51^ This reaction between the tetrazine diene and a strained cyclooctene dienophile is completely bioorthogonal, extremely fast, highly tuneable, and biocompatible (Scheme 3A). Dissociative IEDDA reactions, in which the reaction between 1,2,4,5-tetrazines and *trans*-cyclooctenes (TCOs) enables selective payload release, were developed shortly thereafter (Scheme 3B).^52^ Tetrazines have also been combined with other dienophiles, albeit with some drawbacks owing to poor reaction kinetics and the risk of toxic byproduct formation.^53^

Dissociative IEDDA reactions are one of the most widely used click-to-release reaction types and have shown immense potential for *in vivo* use.^54^ One well-known application is in the context of cancer treatment, where highly selective tumour targeting strategies and local drug release circumvent the severe side effects associated with conventional systemic cancer treatments. This concept was first presented by the group of Robillard, who developed a tumour-recognising antibody functionalised with a TCO-prodrug (so-called ‘antibody drug conjugate’).^52,55^ The drug could be released by adding a tetrazine, leading to release of the potent cytostatic drugs doxorubicin and monomethyl-auristatin E. They demonstrated anti-tumour effects *in vitro* and *in vivo*, with no signs of toxicity or off-target systemic effects.^55,56^ The system was further refined by making tetrazine the antibody-linked prodrug carrier (*via* a secondary amine group on the drug) and using TCO as the releasing agent, resulting in faster (and fully completed) click-to-release reactions *in vitro* and *in vivo*.^57^ Overall, dissociative IEDDA reactions are highly tuneable and enable control over release kinetics even within complex biological systems.

## 5. Click and click-to-release chemistry applied to biomaterials and/or effector molecule delivery

Although the need for robust spatiotemporal release of effector molecules in wound healing is widely acknowledged, the utilisation of the click and click-to-release chemistry chemical toolbox in biomaterials designed for wound healing has remained limited. In the context of wound healing, we previously reported on the use of a bi-functional TCO molecule to achieve spatiotemporal release of growth factors from porous type I collagen scaffolds.^58^ Another group developed an IEDDA-based ‘clickable’ hydrogel with poly(ethylene glycol) (PEG)-norbornene and chondroitin sulfate-tetrazine that showed wound healing potential by supporting wound bed vascularisation in mice.^59^ However, click and click-to-release chemistry reactions have been used in a wide variety of studies on biomaterial production and/or their functionalisation with biologically active molecules. Here, we highlight studies from various research fields that may be translated to biomaterials for wound healing or provide inspiration for their design.

**Fig. 4:**
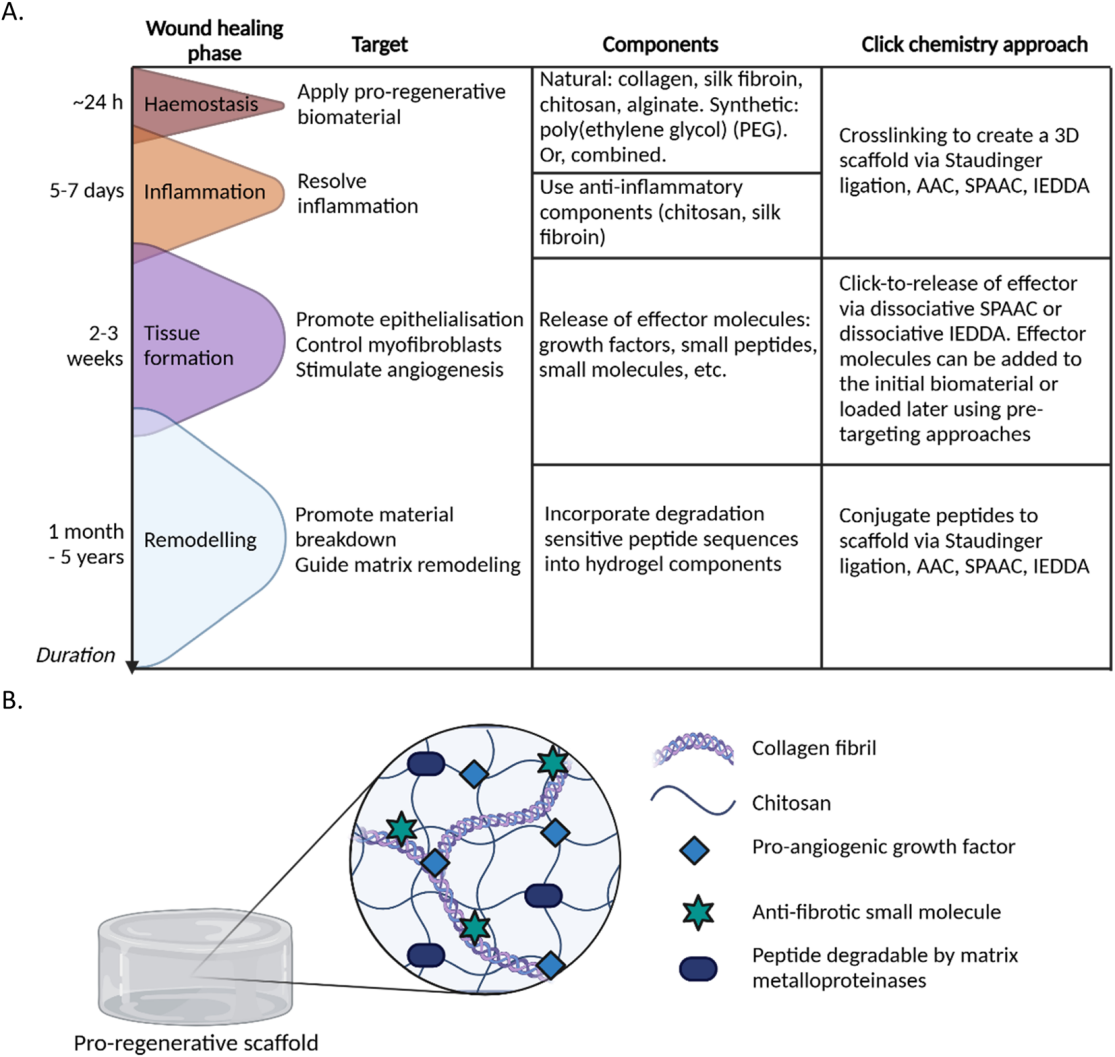
Potential applications of click and click-to-release chemistry in biomaterials for skin regeneration. (A) Table displaying potential targets for the wound healing phases. For each target, multiple components and click chemistry approaches can be employed to make the final pro-regenerative biomaterial. (B) A possible design for a pro-regenerative biomaterial. The scaffold base consists of type I collagen (for structure and biocompatibility) crosslinked with chitosan (antimicrobial, anti-inflammatory) from which a pro-angiogenic growth factor and anti-fibrotic small molecule can be released at different timepoints *via* click-to-release chemistry during the tissue formation phase. Matrix metalloproteinase-sensitive peptides can be added during the initial crosslinking step to improve control over degradability of the scaffold. Created with https://BioRender.com.

This section highlights the versatility of click and click-to-release chemistry, emphasising its adaptability for biomaterial applications. Potential applications in the context of wound healing are proposed throughout, and are summarized in Fig. 4.

### 5.1. Crosslinking and simple functionalisation of biomaterials

Hydrogels are versatile, water-holding polymeric networks that are used in a wide variety of fields, including wound healing. These materials regularly require covalent crosslinking to maintain a specific and stable shape. Crosslinking usually involves toxic chemicals that complicate *in vivo* application. Click chemistry has become an invaluable tool that facilitates crosslinking without toxic additives. Additionally, the polymeric components of hydrogels may be biologically inert, and these can be easily and selectively functionalized with biologically active peptides *via* click chemistry. The examples provided here can be translated to wound-healing applications, as the functionalisation of polymers with click-reaction groups can be performed relatively easily and with readily available materials.

**Scheme 4:**
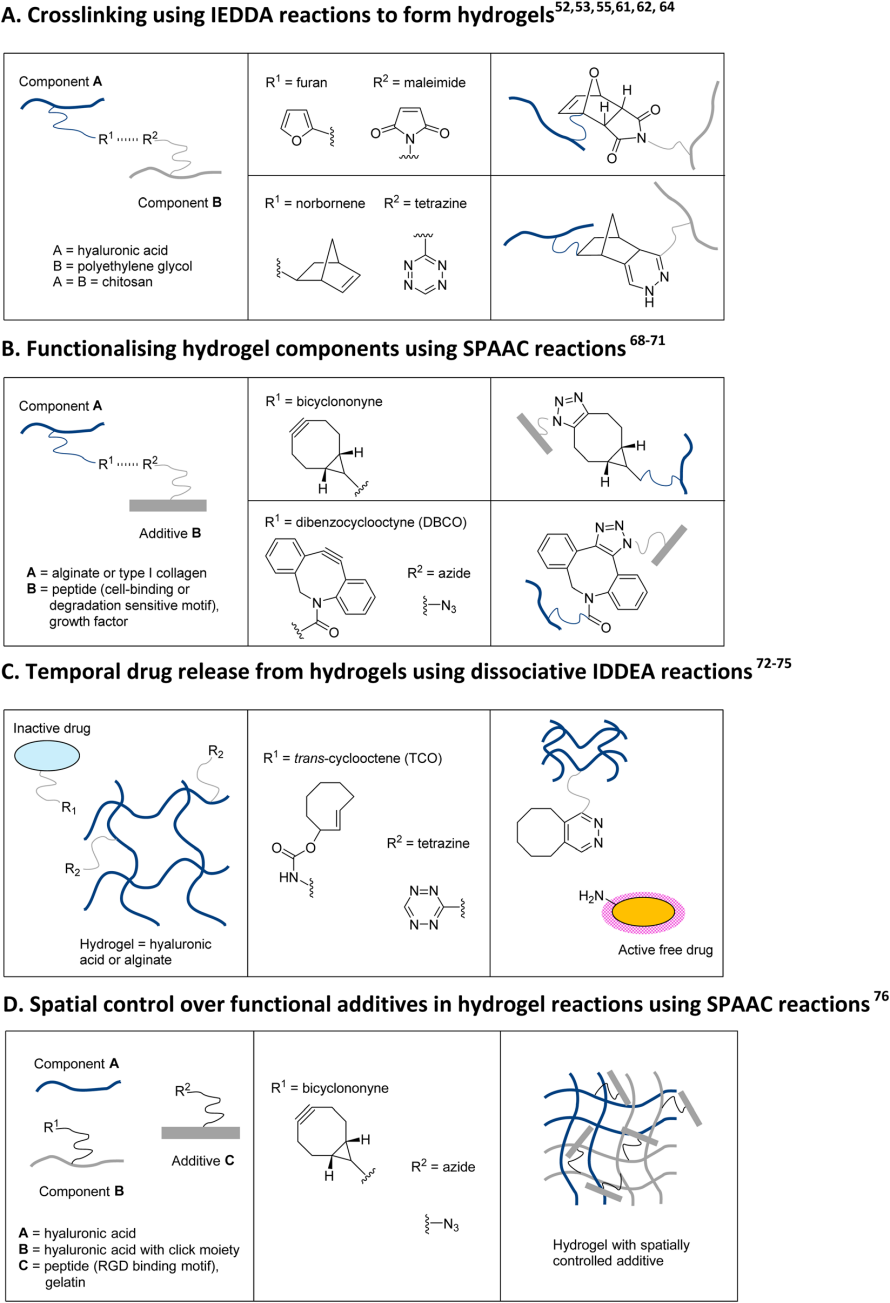
Examples of click and click-to-release reactions in biomaterial production, which have application potential in biomaterials for skin regeneration. (A) Inverse electron demand Diels–Alder (IEDDA) reactions can be used to crosslink hydrogel components without using additional chemical reagents. (B) Hydrogels can be functionalised with bioactive additives, such as peptides or growth factors by using click chemistry moieties that facilitate ligation *via* strain-promoted azide–alkyne cycloaddition (SPAAC) reactions. (C) Dissociative IEDDA reactions can be employed to facilitate temporal control over drug release kinetics. (D) Controlling the spatial distribution of bioactive additives can be achieved *via* hydrogel networks where only certain regions contain the click moiety.

Hyaluronic acids are an important component of the skin's extracellular matrix and are often used in hydrogel as promotors of wound healing.^60^ Hyaluronic acid-based gels are also popular in other fields. For example, researchers have created an injectable hydrogel using hyaluronan functionalised with furan and a PEG-bis-maleimide to achieve crosslinking *via* the Diels Alder cycloaddition (Scheme 4A).^61^ This injectable hydrogel was designed for intrathecal drug delivery to treat spinal cord injury. The gels’ rheological properties could be controlled by adjusting the amount of PEG-bis-maleimide, while the gel was biocompatible *in vivo* as demonstrated using a rat spinal injury cord model. In another approach, the nitrogen released during IEDDA reactions between norbornene-carrying hyaluronic acid and tetrazine-carrying polyethylene glycol resulted in the creation of porous hydrogels that were non-toxic and could slowly release encapsulated curcumin (Scheme 4A).^62^ The porosity, gelation time and mechanical properties could be controlled by varying the ratios of norbornene-to-tetrazine. Chitosan, a natural polymer derived from chitin, is another popular choice due to its antimicrobial properties and positive effects on wound healing.^63^ To circumvent the use of harmful crosslinking reagents and to promote chitosan-based hydrogels for oral drug delivery applications, chitosan polymers were functionalised with maleimide or furan to achieve crosslinking *via* Diels–Alder cycloaddition (Scheme 4A).^64^ The resulting gel was biocompatible with pH-sensitive swelling characteristics, variable pore sizes and could be loaded with the antibiotic drug chloramphenicol. If translated to wound healing applications, the ability to control material properties is highly advantageous as these characteristics can influence cellular responses.^65,66^

Biomaterial characteristics may be further improved by incorporating peptides that promote specific cellular behaviours.^67^ The relatively inert natural polymer alginate is a well-known component in hydrogels for wound healing applications. Functionalisation of alginate with RGD peptides improves the interaction with cells, but incorporation of these peptides *via* chemical crosslinking is unspecific as there is no control over which groups react, and this may reduce the peptides’ reactivity. To overcome these limitations, alginate polymers can be functionalised with the strained cyclooctyne bicyclononyne (BCN) that reacts with azide groups conjugated to cyclic RGD peptides in a SPAAC reaction (Scheme 4B).^68^ This approach improved the bioactivity of the cyclic RGD peptides and promoted cell spreading and proliferation of murine osteoblasts and mesenchymal stem cells. In wound healing applications, the incorporation of RGD peptides may improve biocompatibility. Another interesting option may be to include cleavable peptides to facilitate biomaterial remodelling by invading cells during wound healing. A recent study presented a peptide sequence (proline-valine-glycine-leucine-isoleucine-glycine: PVGLIG) that is sensitive to matrix metalloproteinases and functionalised with azide groups, allowing conjugation to a BCN-functionalized alginate gel *via* SPAAC (Scheme 4B).^69^ Dermal fibroblasts grown in these gels formed a multicellular network, produced their own extracellular matrix (demonstrated by local fibronectin depositions) and over time the gene expression of matrix metalloproteinase 2 increased. The results indicated the PVGLIG-functionalised hydrogels supported remodelling of the material by the embedded fibroblasts.

Larger biomolecules such as growth factors can also be conjugated to biomaterials *via* click chemistry. For example, type I collagen (coated on glass slides) functionalised with a DBCO-sulfo-NHS ester allowed the capture and immobilisation of azide-modified epidermal growth factor (EGF) in a SPAAC reaction (Scheme 4B).^70^ This EGF-functionalised collagen promoted the proliferation of corneal epithelial cells, demonstrating the maintained biological activity of EGF – even when covalently bound – and its biocompatibility. Another study using EGF in the context of corneal wound healing described the preparation injectable hydrogels that crosslinked *via* the SPAAC mechanism.^71^ Here, azide-functionalised collagen reacted with DBCO-PEG-hyaluronic acid (functionalised with UV-reactive photocleavable linkers carrying EGF) to form hydrogels upon injection into corneal defects. In *ex vivo* rabbit and *in vivo* rat models, the UV-triggered release of EGF from the hydrogels improved corneal wound healing compared to PBS and non-releasing hydrogels as the corneal defects closed faster, and the regenerated areas displayed a normal distribution of epithelial differentiation markers.

### 5.2. Achieving spatiotemporal control in biomaterials

The incorporation of temporal characteristics into biomaterials has become relatively straightforward owing to click(-to-release) chemistry. DiMartini and associates reported a two-phase approach for temporal drug delivery in diseased tissues with high concentrations of reactive oxygen species (ROS).^72^ They presented a pre-targeting approach where polyethylene glycol diacrylate (PEGDA) and acrylated-PEG-azide will form a network at sites with high levels of ROS which allowed for localised gelation in diseased tissues. The network could be targeted with a fluorescent reporter (Cy5 or rhodamine B): this reporter also contained a degradation-sensitive peptide sequence with a DBCO moiety. SPAAC reactions between the PEG-azide network and DBCO's resulted in immobilisation of Cy5 or Rhodamine B at the high ROS site. Release of the fluorescent reporter could be temporally controlled by adding collagenase, as this enzyme broke down the peptide sequence between DBCO and reporter. Although only a proof-of-concept study, this approach may be suitable for wound-healing applications as wounds have high levels of matrix metalloproteinases, especially during the remodelling stage. In addition, the wound site is easily accessible – either topically or systemically – which facilitates the administration of various DBCO-coupled effector molecules.

At the time of writing, an IEDDA-based click-to-release drug delivery approach for the treatment of soft tissue sarcoma was ongoing (https://ClinicalTrial.gov ID: NCT04106492). Here, the Oneto and Royzen lab presented a tetrazine-functionalised hyaluronic acid hydrogel, which was injected at the tumour site while a TCO-functionalised doxorubicin prodrug was injected systemically, leading to local doxorubicin release and resulting in effective anti-tumour treatment in mice (Scheme 4C).^73,74^ As mentioned, hyaluronic acid-based injectable gels are good candidates in wound healing applications owing to their biocompatibility and easy administration. Moreover, serial administration of various regeneration-stimulating prodrugs may facilitate easy temporal control. A similar drug delivery system was proposed by Czuban and colleagues from the Oneto and Royzen lab to improve the delivery of antibiotics (Scheme 4C).^75^ They used an IEDDA reaction pair consisting of a tetrazine-modified alginate gel, which can be injected at the infection site, and a systemic injection of TCO-modified prodrugs. Multiple doses of antibiotics could be activated *in vitro*, and the released drugs inhibited biofilm formation by bacteria. The approach was also tested in a mouse model, where the bacterial load in the tissue could be reduced by injecting the tetrazine-alginate gel at the infection site followed by a systemic injection of TCO-prodrug. As alginate is generally applied in wound healing products, the latter example is highly adaptable.

Spatial control over effector molecules remains more challenging. The previous examples lean more towards temporal control, with the spatial aspect being the result of site-specific injection or gelation cues. Robust spatial control over material characteristics may be achieved by bioprinting. Here, 3D printers allow for the combination of (multiple) biomaterials with control over the spatial characteristics within one construct. Building upon this approach, Tournier and associates introduced a 4D bioprinting concept (‘clickable dynamic bioinks’) (Scheme 4D).^76^ A biocompatible ink was developed using hyaluronic acid (HA), which contained moieties to enable boronate-ester crosslinking. Changing the HA content and the percentage of substituted (boronate-ester) groups provided control over the hydrogel characteristics, including stiffness and swelling behaviour. HA could be functionalised with BCN, which yielded hydrogels that could be modified post-printing with azide-carrying polymers and peptides (such as RGD cell binding motifs or gelatine). Spatial control over these post-printing modifications could be achieved by interchanging BCN-functionalised and non-functionalised HA inks during the printing process. In this way, the BCN click moieties – and any peptides/polymers added – could be sequestered to a specific location within the material. This proof-of-concept study highlights the tuneability of a bioprinting-based approach and could assist in creating more complex materials for wound healing applications.

### 5.3. Increasing complexity and controlling drug release kinetics

Controlling effector molecule activity is key to achieving robust spatiotemporal signalling. As the previous section highlighted, this can be achieved by the subsequent administrations of compounds. For all approaches where temporal control is desired, it is essential that the bound payload remains inactive until released. For effector molecules that require uptake by cells to be active this is not a major obstacle. However, it may be challenging to achieve this with effector molecules that bind receptors on the cell surface, such as growth factors. A method to safeguard inactivity of the effector molecule would be to control its orientation and ensure the active binding sites are inaccessible. A study aiming for the opposite effect, where homogeneous activity of bone morphogenic protein 2 (BMP2) was desired, may offer inspiration. The researchers created BMP2 containing an artificial amino acid (propargyl-l-lysin) that ensured that BMP2, when coupled to azide-functionalised agarose beads *via* CuAAC, was always positioned with its active binding site exposed.^77^ Turning this system around and ensuring the active sites are covered when the growth factor is ligated to its carrier would enable temporal control over its activity.

Effector molecule delivery may be further controlled by combining multiple click-reaction types or adapting specific reaction parameters to specific needs. A method to induce sequential release of two separate effector molecules was described by Robertson and associates.^78^ They designed a TCO molecule with two orientations to achieve fast release within 1 hour (di-axial orientation) or slow release over 96 hours (di-equatorial orientation) of two drugs from nanoparticles upon one single administration of the tetrazine activator. This ‘staggered’ release approach was successful in administering the chemotherapeutic drugs doxorubicin and PAC-1 in triple-negative breast cancer cells, reducing their viability more effectively compared to single drug administrations. Such an approach would allow a material to mimic the physiological signalling processes during wound healing.

So far, most studies have focussed on one single type of click reaction which limits the complexity that can be achieved within one material. Due to their bioorthogonal nature, click reactions can be combined to increase the functionality of a material. Four click chemistries have been combined in one system.^79^ Using a combination of SPAAC, strain-promoted alkyne–nitrone cycloaddition (SPANC), Staudinger–Bertozzi ligation (SBL) and a perfluoroaryl azide Staudinger reaction (PFAA-SR), researchers were able to conjugate and release various molecules from gold nanoparticles. This work demonstrates the complexity that can be achieved within one single system if designed correctly.

## 6. Conclusions

The wound healing phases must be controlled to achieve skin regeneration, but a biomaterial capable of spatiotemporal delivery of appropriate effectors does not exist yet. In this review we provided an overview of key papers employing click and click-to-release chemistry that may be translated to wound healing applications. Selection of the appropriate biomaterial base, effector molecules and click chemistry approach will be dependent on which parts of the wound healing phase are targeted. In this review, we set out to make readers aware of the complicated nature of wound healing and the potential of click chemistry to aid in achieving skin regeneration. The bioorthogonal click chemistry toolbox is ever expanding, providing a myriad of options for researchers to select from. It has made robust spatiotemporal release of effector molecules from biomaterials an accessible goal.

## Author contributions

Conceptualisation: MG, MvdW, WFD. Writing – original draft: MvdW, MG. Writing – review & editing: MG, MvdW, TJB, FPJTR, THvK, WFD. Visualisation: MG, TJB, FPJTR. Supervision: THvK, WFD. Funding acquisition: WFD.

## Conflicts of interest

The authors have nothing to declare.

## Data Availability

No primary research results, software or code have been included and no new data were generated or analysed as part of this review.
